# Regulatory evolution and service delivery models of telepharmacy in Thailand: a review of pandemic responses and future policy directions

**DOI:** 10.1080/20523211.2026.2731648

**Published:** 2026-09-21

**Authors:** Patama Tapchaisri, Anuchai Theeraroungchaisri

**Affiliations:** a Pharmacy Unit, Hospital for Tropical Diseases, Faculty of Tropical Medicine, Mahidol University, Bangkok, Thailand; b Faculty of Pharmaceutical Sciences, Kasetsart University, Bangkok, Thailand

**Keywords:** Thailand, telepharmacy, pharmacy service, COVID-19, healthcare innovation, pharmaceutical policy

## Abstract

**Background:**

The COVID-19 pandemic accelerated digital health adoption in Thailand, disrupting traditional healthcare delivery and prompting rapid pharmaceutical system reforms. Prior to 2020, telepharmacy was virtually non-existent due to strict regulations mandating in-person dispensing at licensed premises; however, the public health crisis prompted policy changes, leading to regulatory relaxation and the formal establishment of telepharmacy standards. To understand these transformative shifts, this review examines the regulatory evolution of telepharmacy in Thailand, classifies diverse service delivery models implemented across hospital and community pharmacy settings, and synthesises effectiveness evidence to inform future pharmaceutical policy and sustainable system integration.

**Methods:**

An integrative narrative review using documentary analysis of official policy and regulatory documents was conducted. Studies evaluating telepharmacy or medication delivery services were identified through PRISMA-guided searches of Thai-Journal Citation Index (TCI), PubMed, and Google Scholar (2020–2025).

**Results:**

Thailand implemented a phased policy response progressing from emergency regulatory flexibility toward institutionalised governance. Seven hospital-based telepharmacy models were identified. Synthesis of 31 included studies (14 pharmacist-led and 17 telemedicine with medication delivery or medication delivery without pharmacist counselling studies) suggested that telepharmacy and medication delivery services generally supported continuity of care for chronic diseases. Studies involving pharmacist interventions more frequently reported favourable outcomes, although findings varied across service models. Implementation relied largely on social media platforms and proprietary applications, though independent pharmacies faced financial barriers to accessing certified platforms.

**Conclusion:**

While telepharmacy has been integrated into routine care under the Universal Coverage Scheme (UCS), an institutionalisation gap remains regarding sustainable public financing and digital infrastructure. Addressing regulatory clarity, reimbursement mechanisms, digital equity, and platform interoperability will be essential for the long-term and equitable integration of telepharmacy.

## 1. Background

The coronavirus disease 2019 (COVID-19) pandemic, which emerged in late 2019, led to disrupted health systems across many countries. This prompted the World Health Organization (WHO) to declare a global pandemic on 11 March 2020 (WHO, 2020). By early November 2021, more than 246 million confirmed COVID-19 cases had been reported worldwide, with approximately 5.0 million COVID-19-related deaths. In Thailand, the first confirmed COVID-19 case was reported in early 2020, and a total of 1,920,189 confirmed cases had been documented by 1 November 2021, ranking the country third in Southeast Asia, following India and Indonesia (Thitichai & Taweekijkarn, 2021). The pandemic has affected economic and social programs (Clemente-Suárez et al., 2021), educational systems (Pokhrel & Chhetri, 2021), and healthcare infrastructure (Clemente-Suárez et al., 2021) worldwide. In Thailand, as in many countries, healthcare services were required to rapidly adapt not only in response to the COVID-19 pandemic but also to ongoing structural pressures within the health system, including population ageing, healthcare workforce constraints, and persistent geographic inequities in access to pharmaceutical care. These converging challenges accelerated the adoption of alternative service delivery models and the increasing reliance on digital health technologies, including telehealth.

Telehealth refers to the use of information and communication technologies to deliver healthcare services remotely, enabling patients to access care from their homes (Basu et al., 2023), and has emerged in many countries (Monaghesh & Hajizadeh, 2020). Within this context, telepharmacy, as a subset of telehealth, refers to the provision of pharmaceutical care by licensed pharmacists through telecommunication technologies (Unni et al., 2021). Although telehealth and telepharmacy services have existed internationally for years, their adoption remained limited in both high-income and low- and middle-income countries prior to the COVID-19 pandemic (Kissi et al., 2023). The COVID-19 pandemic substantially accelerated the adoption and scaling of telemedicine and telepharmacy models (Mahmoud et al., 2022).

Similarly, in Thailand, telepharmacy was virtually non-existent prior to 2020, as pharmaceutical regulations required in-person dispensing by pharmacists at licensed premises. The COVID-19 pandemic created a critical opportunity for policy change, prompting regulatory relaxation and the rapid introduction of formal telepharmacy standards. Implemented across both hospital-based and community pharmacy settings, telepharmacy emerged as a vital mechanism for maintaining medication access for patients with chronic diseases. This approach simultaneously alleviated hospital congestion and minimised unnecessary face-to-face contact.

Following these regulatory changes, telepharmacy services in Thailand have expanded across diverse care settings, supported by digital platforms and medication delivery systems. Although several studies in Thailand have examined specific aspects of telepharmacy, including clinical outcomes, patient satisfaction, and system development, no comprehensive review has yet systematically synthesised regulatory adaptations, service delivery models, and clinical evidence within a single analytical framework. Moreover, the transition from crisis-driven innovation to routine healthcare services has revealed structural challenges. While existing frameworks facilitated the initial integration of telepharmacy within Thailand's publicly funded system, critical policy gaps persist regarding long-term sustainability. In particular, the lack of defined reimbursement mechanisms for digital infrastructure and medication delivery logistics remains a significant barrier to permanent adoption, despite demonstrated clinical and operational benefits. This raises an important policy question regarding how regulatory reforms can be effectively aligned with sustainable financing to support the long-term integration of telepharmacy into routine care.

Accordingly, this review examines the regulatory evolution of telepharmacy in Thailand, categorises service delivery models across hospital and community pharmacy settings, and synthesises effectiveness evidence. The analysis provides policy-relevant insights to support the long-term sustainability of telepharmacy within routine healthcare systems.

## 2. Methods

This integrative narrative review synthesises heterogeneous evidence on the regulatory evolution of pharmaceutical policy and practice in Thailand, with a particular focus on the emergence of telepharmacy in the context of the COVID-19 pandemic. The review integrates multiple sources of evidence, including regulatory and policy documents, descriptions of telepharmacy service delivery models, and studies reporting clinical outcomes. A PRISMA-guided approach was applied to the identification and selection of clinical outcome studies to enhance transparency in the review process. A full systematic review was not undertaken due to substantial heterogeneity in study designs, intervention models, and outcome measures across telepharmacy studies in Thailand, as well as the need to integrate policy and regulatory evidence that is not amenable to conventional systematic review methodologies.

### 2.1 Search strategy

A structured search strategy was used to identify both documentary and clinical evidence sources. For the policy and regulatory component, documentary sources were identified from official governmental repositories, including the Royal Gazette, and the official websites of the Ministry of Public Health, the Food and Drug Administration of Thailand, and the Pharmacy Council of Thailand. Documentary sources included laws and regulations governing pharmaceutical practice, Royal Gazette notifications, official announcements, professional standards related to telepharmacy, curriculum materials, and technical guidelines from the Telepharmacy Practitioner Training Program developed by the Pharmacy Council of Thailand. Additional reports from academic and governmental organisations, together with relevant peer-reviewed studies, were also reviewed for their relevance, authority, and contribution to telepharmacy implementation and policy development in Thailand. Documents unrelated to telepharmacy and superseded regulatory documents were not considered.

**Figure 1.**
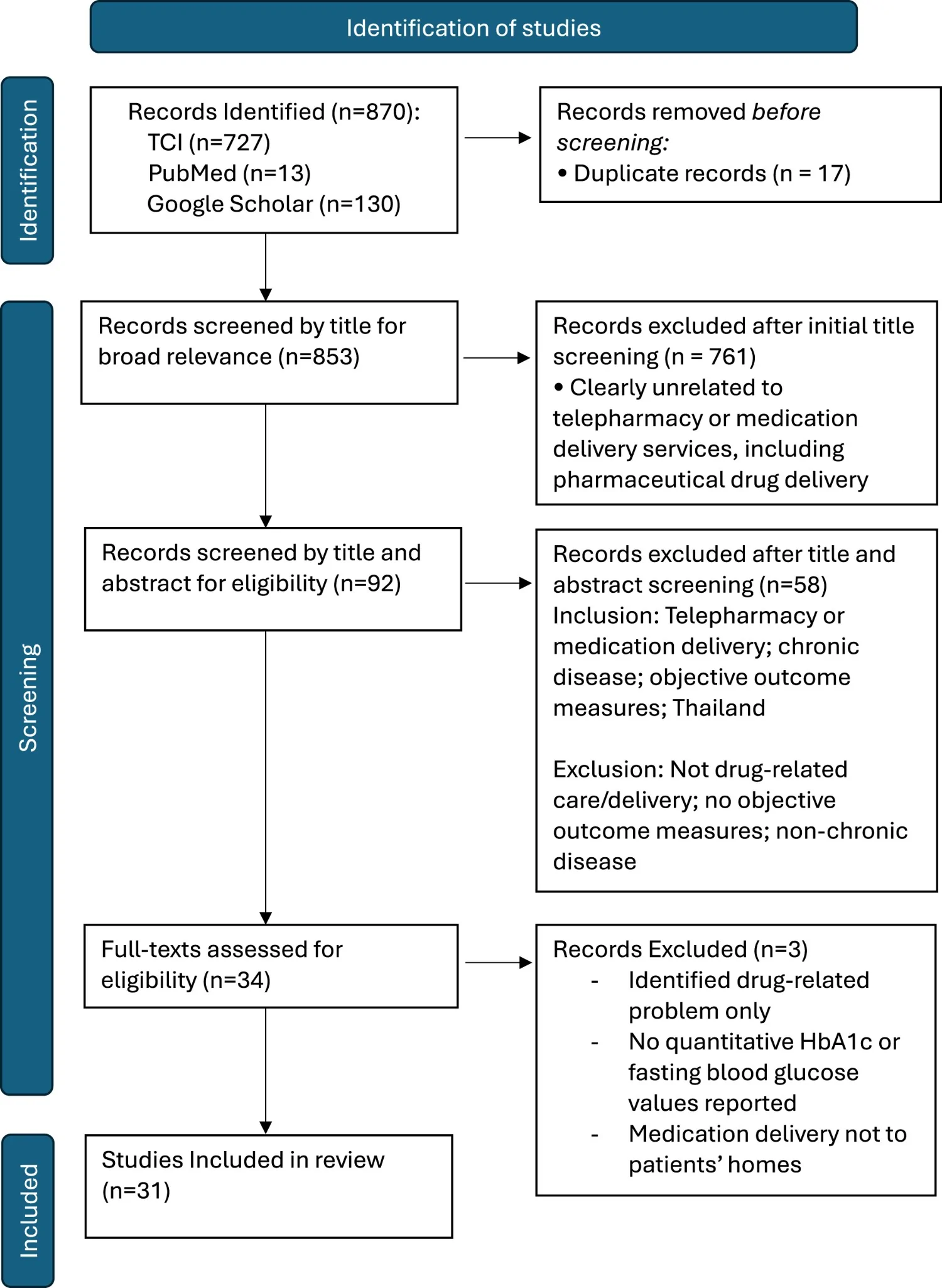
PRISMA flow diagram for study identification and selection process.

For the clinical evidence component, the identification and selection of studies evaluating the effectiveness of telepharmacy or medication service delivery were conducted in accordance with the PRISMA 2020 guidelines (see Figure 1 for PRISMA diagram). Literature searches were conducted in January 2026 in the Thai-Journal Citation Index Centre (TCI), PubMed, and Google Scholar to identify studies published in English or Thai between 2020 and 2025. Search terms included combinations of ‘telepharmacy’, ‘medication delivery’, or ‘remote’ with ‘Thailand’ or ‘Thai’, together with pharmacist-related terms. The detailed search strategies for each database are provided in Table 1. Following duplicate removal, records underwent an initial title-level screening for broad relevance, followed by title and abstract screening of potentially relevant records against the eligibility criteria. Studies were included if they evaluated outcomes relevant to the effectiveness of telepharmacy or medication service delivery in Thailand, including clinical, medication-related, healthcare utilisation, and economic outcomes. Studies reporting only patient satisfaction or behavioural outcomes without objective outcome measures relevant to telepharmacy or medication delivery services were excluded.

**Table 1.** The detailed search strategies for each database.

Database	Exact search terms	Search date	Language	Screening approach
TCI	Separate searches using ‘telepharmacy’, ‘ส่งยา’ (medication delivery), and ‘ทางไกล’ (remote)	11 Jan 2026	Thai	Title/abstract screening followed by full-text assessment
PubMed	((‘medication delivery’[tiab] OR ‘medicine delivery’[tiab] OR ‘pharmacy delivery’[tiab] OR ‘home delivery’[tiab] OR telepharmacy[tiab]) AND (Thailand[tiab] OR Thai[tiab])) NOT (nanoparticle[tiab] OR liposome[tiab] OR polymer[tiab] OR ‘drug delivery system’[tiab])	22 Jan 2026	English	Title/abstract screening followed by full-text assessment
Google Scholar	(‘telepharmacy’ OR ‘pharmacy delivery’ OR ‘medication delivery’) AND (Thailand OR Thai) AND (pharmacist OR pharmacy) – nanoparticle – liposome – targeting – education	22 Jan 2026	English & Thai	Relevant records identified by title/abstract screening followed by full-text assessment.

Note: TCI is an abbreviation for the Thai-Journal Citation Index Centre, a database system that tracks and evaluates academic journals published in Thailand.

### 2.2 Data extraction and synthesis

For the policy and regulatory component, data extracted from documentary sources included document type, issuing organisation, year of publication, regulatory scope, and key policy content. These data were synthesised thematically into four analytical domains:

1. Regulatory and legal framework adaptations for telepharmacy
2. Policy-driven development and innovation in telepharmacy
3. Telepharmacy service delivery models in hospital and community pharmacy settings
4. Pharmacist roles and policy-relevant outcomes in telepharmacy practice.

For the clinical evidence component, data extracted from included studies comprised study design, care setting, population, intervention characteristics, healthcare professional involvement, comparator (where applicable), and outcomes relevant to the effectiveness of telepharmacy or medication delivery services. The findings were synthesised narratively because of substantial heterogeneity in study designs, interventions, and outcome measures. Formal risk-of-bias assessment was not undertaken because this review was not designed as a systematic review. Instead, methodological characteristics and commonly reported limitations across studies were systematically examined and considered when interpreting the findings.

## 3. Results

### 3.1 Regulatory and legal evolution of telepharmacy in Thailand

In Thailand, pharmaceutical regulation is governed by two primary organisations: the Thai Food and Drug Administration (Thai FDA), which regulates pharmaceutical products from a legal perspective, and the Pharmacy Council of Thailand, which is responsible for professional accreditation and the regulation of pharmacists’ professional practice. Prior to 2020, telepharmacy did not exist as a legally recognised service in Thailand, as the Thai FDA required patients to receive pharmaceutical consultations and medications exclusively at physical pharmacy locations. This requirement was reinforced by Section 19(1) of the Thai Drug Act B.E. 2510 (1967) (Government of Thailand, 1967), which prohibits drug sales outside licensed premises except for wholesale distribution. However, some hospitals implemented medication delivery by mail for patients who had already received proper counselling and assessment from pharmacists at the counter. This service was primarily available to patients with stable conditions to reduce congestion and minimise waiting times for medication (Division of Pharmacy, Siriraj Hospital, 2020). Unlike hospitals, community pharmacies were strictly prohibited from providing online medication sales or home delivery services. These restrictions were intended to prevent risks such as counterfeit or substandard medications, exaggerated advertising claims, and improper labelling. They also aimed to reduce the risk of medication misuse in the absence of pharmacist supervision, which could lead to harmful outcomes such as drug interactions or adverse drug events (Government of Thailand, 1967).

The turning point for telepharmacy in Thailand occurred during the COVID-19 pandemic, when government-imposed lockdown measures, including nationwide travel restrictions and physical distancing requirements, led to the adoption of telemedicine instead of on-site hospital visits (Zayar et al., 2024).

On 2 June 2020, the first Notification of the Pharmacy Council of Thailand on the Establishment of Standards and Procedures for Telepharmacy Services was issued by the Pharmacy Council of Thailand to enhance access to pharmaceutical care through telepharmacy (Pharmacy Council of Thailand, 2020). This regulatory shift prompted hospitals to modify their pharmaceutical services by adopting remote counselling and medication delivery, while community pharmacies began offering remote consultations and home medication delivery to comply with infection control measures and reduced hospital visits. Collectively, these changes contributed to a broader transformation of the national pharmaceutical system. In terms of financing, Thailand operates three main public health insurance schemes under Universal Health Coverage (UHC): the Universal Coverage Scheme (UCS), the Civil Servant Medical Benefit Scheme (CSMBS), and the Social Security Insurance (SSI) scheme (Marshall et al., 2021). Approximately 75% of the population is covered by the UCS (Paek et al., 2016). Medication delivery fees are covered for beneficiaries under the Universal Coverage Scheme (UCS), whereas beneficiaries under the Civil Servant Medical Benefit Scheme (CSMBS), the Social Security Insurance (SSI) scheme, and self-pay patients generally pay a nominal fee for medication delivery (The Coverage, 2024). During the outbreak of the COVID-19 Alpha and Delta variants in early 2021, the Ministry of Public Health reported that between 11 April 2020 and 9 January 2021, a total of 176,924 medication deliveries were made by mail from 217 hospitals. Among these, hypertensive patients represented the largest group utilising the service, accounting for 41,999 deliveries (Ministry of Public Health Contact Center, 2021).

Nevertheless, the Pharmacy Council of Thailand undertook continuous regulatory adjustments to ensure alignment with the national healthcare context, existing service infrastructures, and evolving public health needs. In early 2022, a series of multi-stakeholder forums were convened to develop regulatory frameworks for telepharmacy and online pharmacy services. These forums engaged public and private sector stakeholders, healthcare professionals, academic institutions, service users, and healthcare purchasers, and focused on establishing service standards, platform selection criteria, workforce competencies, public health literacy, and mechanisms to ensure public access and service verification. On 6 July 2022, the Pharmacy Council of Thailand issued the updated Regulation of the Pharmacy Council of Thailand on Restrictions and Conditions for the Practice of Pharmacy Profession to support the implementation of telepharmacy standards. The regulation defines ‘telepharmacy’ as a pharmaceutical service encompassing medication consultation, identification, prevention, and resolution of medication-related problems, medication safety monitoring, and other related pharmacy services delivered to recipients through telecommunication technologies. These services must be provided by licensed pharmacists practicing in government service units, healthcare facilities, or community pharmacies. Key requirements for telepharmacy services include provider and patient registration; secure digital record-keeping and medication monitoring; standardised service systems, models, and operational procedures; specified categories of medications eligible for telepharmacy services; and secure medication delivery (Secretariat of the Cabinet, 2022a). This regulatory framework was further operationalised through the Notification of the Pharmacy Council of Thailand on Guidelines for Telepharmacy Service Standards, issued on 30 August 2022 (Pharmacy Council of Thailand, 2022). Under this notification, pharmacists providing telepharmacy services are required to complete and pass a qualifying examination following a comprehensive telepharmacy training curriculum developed by the Pharmacy Council of Thailand. This free online training program aims to strengthen pharmacists’ competencies in relevant laws and regulations, telepharmacy platform utilisation, digital health tools, and compliance with the Personal Data Protection Act (PDPA) (Pharmacy Council of Thailand, 2023a).

Moreover, licensed pharmacists are required to deliver telepharmacy services through applications certified by the Pharmacy Council of Thailand. During remote consultations, pharmacists must adhere to professional and ethical standards by evaluating prescription appropriateness, identifying potential drug-related problems (e.g. drug interactions and inappropriate medication use), and providing appropriate interventions as needed. This includes established protocols for referral to healthcare facilities when cases exceed the pharmacist's scope of practice, as well as systems for follow-up and continuity of care. To support these professional requirements, telepharmacy systems are required to demonstrate at least the following functional capabilities (Pharmacy Council of Thailand, 2022).

- **Provider registration and verification:** Authentication of the pharmacist's name, professional license number, and service location.
- **Patient identity verification:** Confirmation of patient identity and collection of necessary health information.
- **Secure digital records and medication monitoring:** Explicit patient consent and maintenance of comprehensive digital records, including patient history, service details, and medication monitoring data, in compliance with relevant data protection laws, including the Personal Data Protection Act B.E. 2562 (A.D. 2019), the Electronic Transactions Act B.E. 2544 (A.D. 2001), and the Cybersecurity Act B.E. 2562 (A.D. 2019).
- **Multimedia communication support:** Facilitation of remote service delivery through video, audio, and text-based communication.
- **Secure medication dispensing and delivery:** Safe storage, packaging, and transportation of medications, including tracking and confirmation mechanisms to ensure timely and accurate delivery to intended recipients.

**Figure 2.**
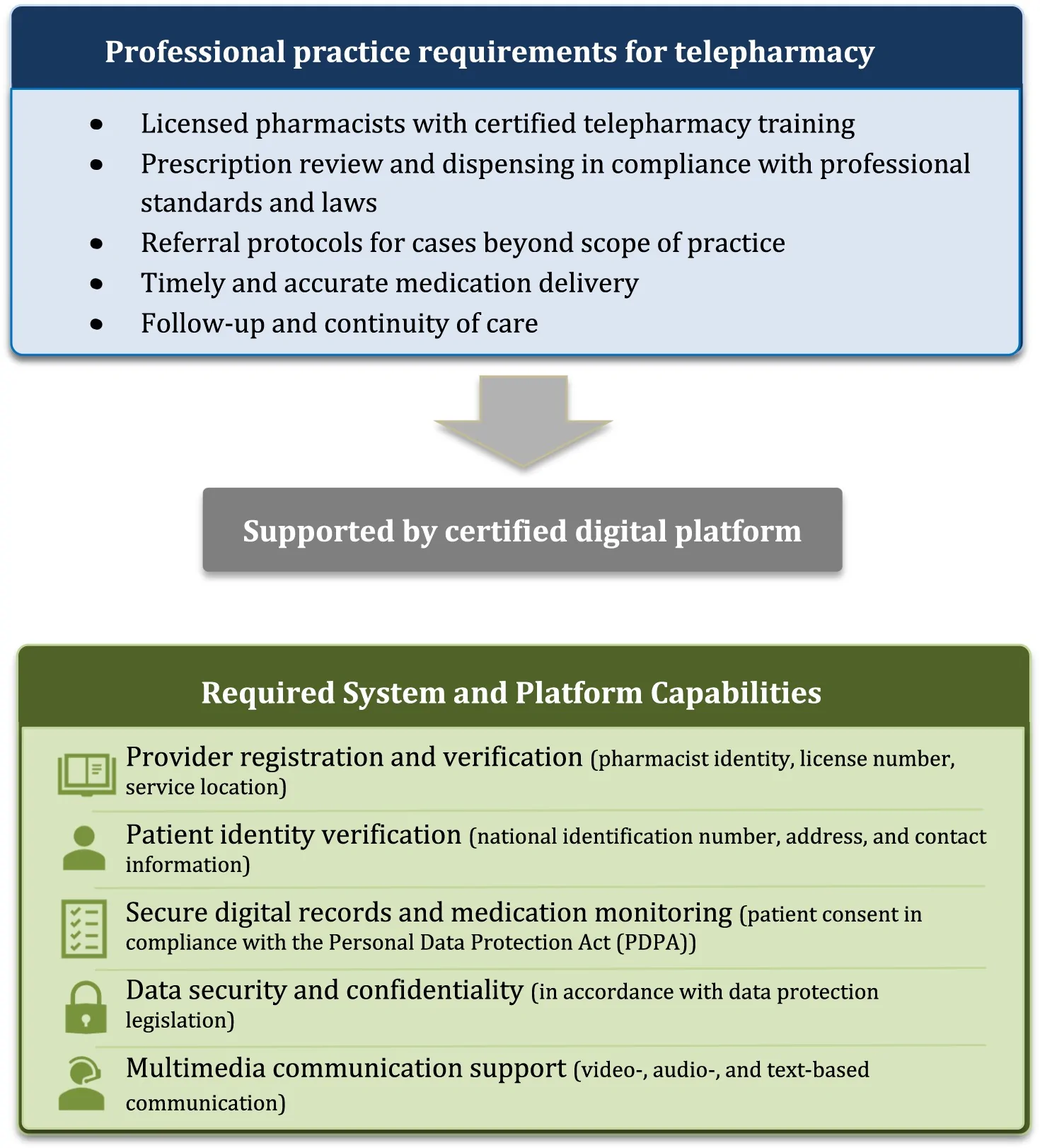
Regulatory framework for telepharmacy services in Thailand.

Figure 2 summarises the professional practice requirements and system and platform capabilities governing telepharmacy services in Thailand.

With these regulations in place, mobile applications and social media platforms have become key tools for telecommunication services due to their accessibility and convenience. In Thailand, various social media platforms, particularly LINE Official Account, are widely used. In hospital settings, these platforms are commonly utilised as communication channels between healthcare professionals and patients (Line for Business, 2021). In parallel, many hospitals have developed proprietary mobile applications to deliver telecommunication services to patients (HealthServ, 2024).

In community pharmacy settings, LINE Official Account is also widely used as a communication tool. However, large chain pharmacies in Thailand typically have greater financial capacity to invest in proprietary telepharmacy platforms, such as the ALL PharmaSee application used by Exta Plus pharmacies under CP ALL Public Company Limited (Pharmacy Council of Thailand, 2024), and the BangkokDrugstore application developed by Bangkok Drugstore Company Limited (Pharmacy Council of Thailand, 2023b). Independent pharmacies, by contrast, often face financial constraints that limit their ability to develop and maintain certified digital platforms. To address this gap, the Pharmacy Council of Thailand developed a centralised application, TeleHealth Thailand, which meets the Council's regulatory requirements and provides independent pharmacies with free access to essential telepharmacy tools. This initiative aims to enhance access to public healthcare services and support healthcare professionals across community pharmacies, hospitals, and other healthcare settings. Through the TeleHealth Thailand platform, patients can locate nearby pharmacies and receive video consultations from pharmacists. The platform also offers additional features, including online clinics and health-related educational content. Planned enhancements include integrated payment systems linked to health insurance coverage and improved interoperability with other healthcare applications. The platform is continuously updated and maintained by the Pharmacy Council's development team (Lertsinudom, 2025).

**Figure 3.**
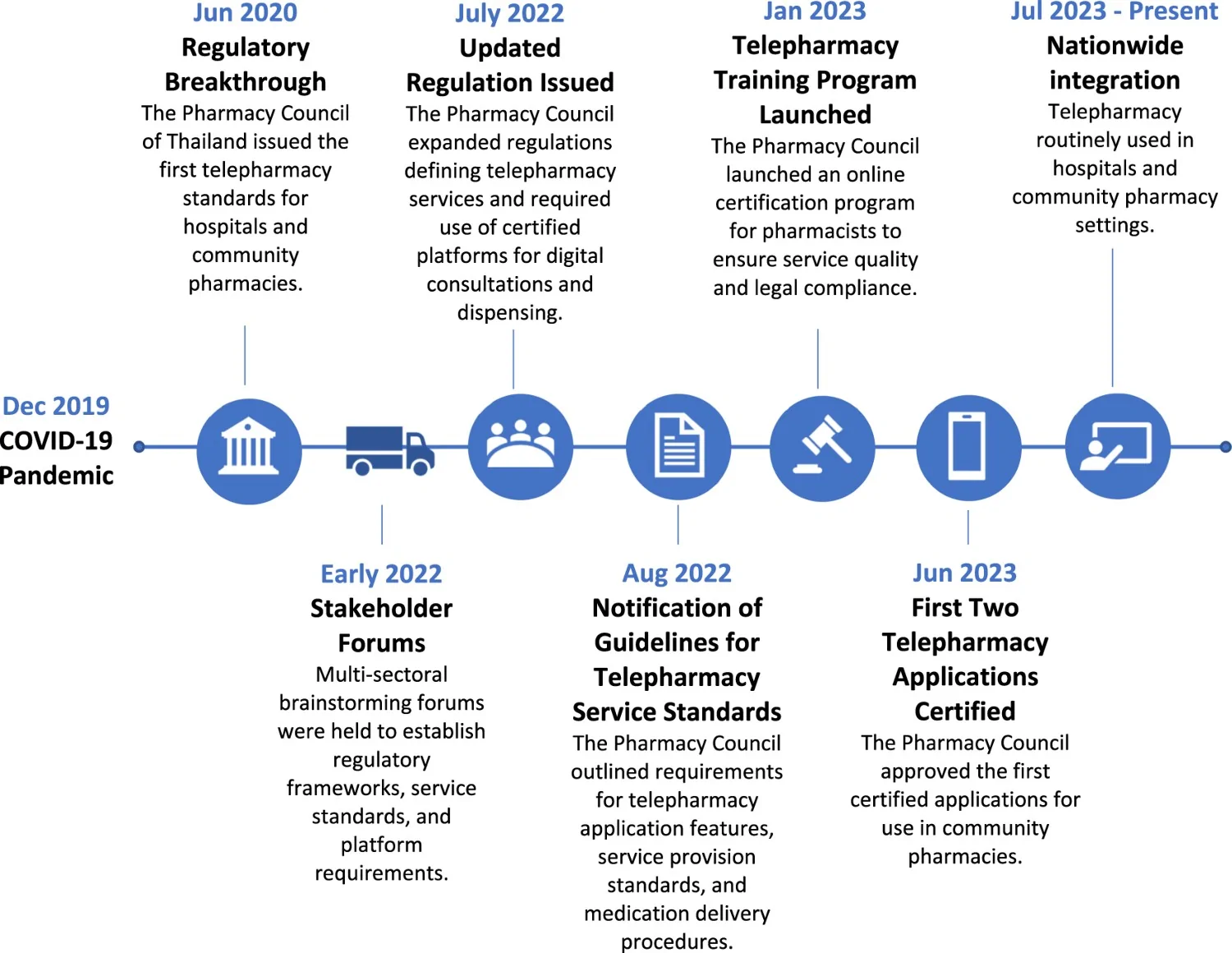
Timeline of telepharmacy development in Thailand.

Although COVID-19 was reclassified as a monitored communicable disease on 1 October 2022 – similar to influenza (Secretariat of the Cabinet, 2022b) – telepharmacy services have continued to be widely used and are undergoing further development, particularly in digital health applications. The historical timeline of telepharmacy development in Thailand is summarised in Figure 3.

### 3.2 Telepharmacy service delivery models

#### Service delivery models in hospitals

Between January and February 2023, a national cross-sectional descriptive study conducted by Chiang Mai University surveyed 421 hospitals under the Ministry of Public Health (Singrungruangkij et al., 2024). The findings, based on data from 165 hospitals (39.19%), have developed telepharmacy services, mostly found in specialised hospitals (28 hospitals) and large general hospitals (18 hospitals). A total of seven telepharmacy service models were observed. These models were grouped into two categories: (1) telepharmacy services integrated with medical care and (2) telepharmacy services offered independently. However, each hospital may provide more than one model.

##### Category 1: Telepharmacy with medical services

**Model 1: Telemedicine with telepharmacy**

Patients receive medical consultations via telemedicine, followed by medication counselling through telepharmacy. Medications are then delivered directly to their homes, a service utilised by 52.12% of hospitals surveyed (*n* = 86).

**Model 2: Hospital treatment with home medicine delivery**

Patients receive in-person medical services at hospitals, then are enrolled in a Home Medicine Delivery Program. Pharmacists provide telepharmacy consultations before medications are sent to patients’ homes, a service utilised by 39.39% of hospitals surveyed (*n* = 65).

**Model 3: Telemedicine and telepharmacy with Sub-district Health-Promoting Hospitals (SHPHs)**

Patients receive medical consultation via telemedicine, followed by medication consultation through telepharmacy. Prescribed medicines are delivered to SHPHs for patient pickup or home delivery facilitated by Village Health Volunteers (VHVs). This model was utilised by 30.91% of surveyed hospitals (*n* =   51).

**Model 4: Hospital-based medical services with telepharmacy and SHPHs**

Patients receive in-person medical services at hospitals and are subsequently registered for the SHPH Medicine Delivery Program. Medication consultations are then provided by pharmacists through telepharmacy. Prescribed medicines are delivered to SHPHs for patient pickup or home delivery facilitated by VHVs. This model was utilised by 25.45% of surveyed hospitals (*n* =   42).

**Model 5: Hospital-based medical services with community pharmacy**

This model involves collaboration between hospitals and community pharmacies. Patients receive in-person medical services at hospitals, with prescribed medicines prepared and delivered through community pharmacies using one of three approaches: (1) medicines prepared by hospital pharmacists and transferred to community pharmacies; (2) dispensing from hospital-supplied pharmaceutical stock at community pharmacies; or (3) dispensing from community pharmacy inventory. Medication consultations are provided by community pharmacists through telepharmacy, followed by home delivery of medicines. This model was utilised by 10.30% of surveyed hospitals (*n* =   17).

##### Category 2: Telepharmacy without medical services

**Model 6: Telepharmacy for consultation and monitoring**

Patients receive medication consultation and drug use monitoring from pharmacists through telepharmacy, including medication adherence, adverse drug reaction monitoring, and health promotion activities. This model was utilised by 35.15% of surveyed hospitals (*n* = 58).

**Model 7: Telepharmacy for chronic disease patients requiring medication refills**

Designed for patients needing medication refills, this model supports ongoing monitoring and consultation without medical visits. Medications are delivered to patients’ homes after pharmacist review, utilised by 30.91% of hospitals surveyed (*n* = 51).

**Figure 4.**
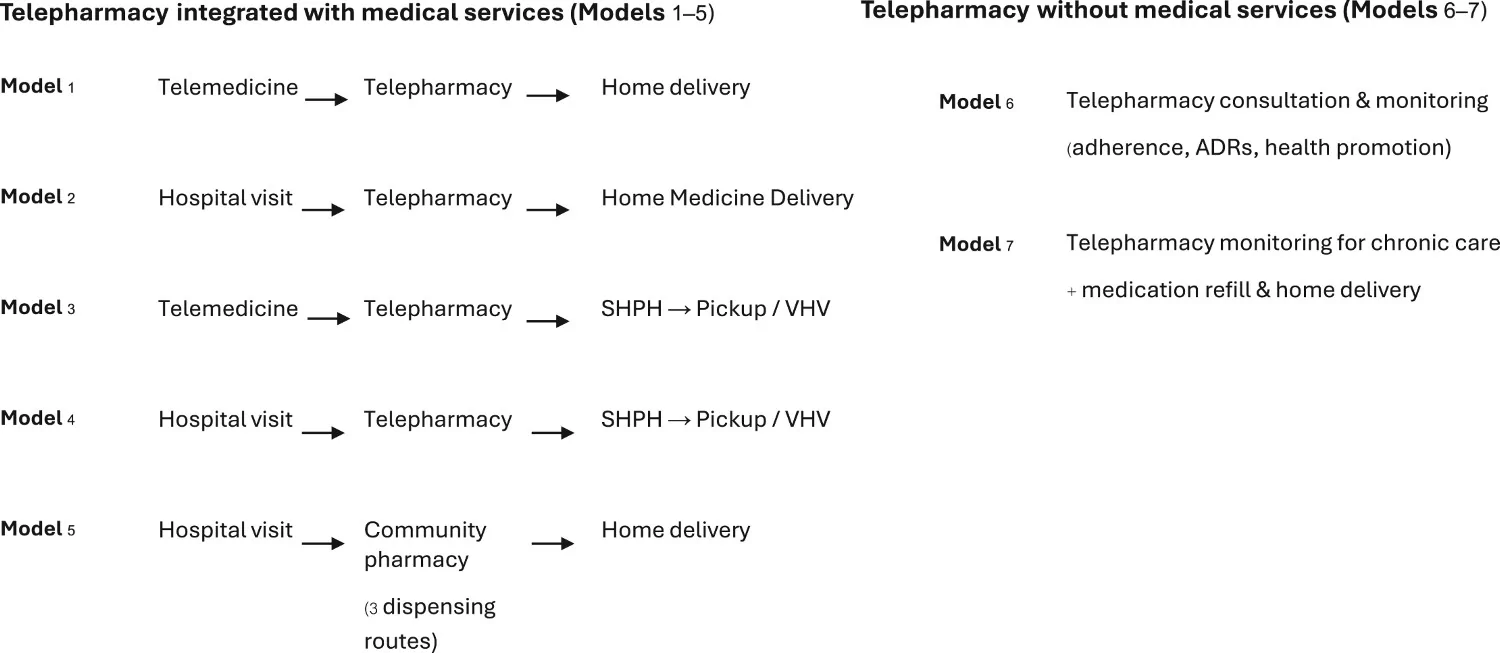
Hospital-based telepharmacy service delivery models in Thailand (27).

The hospital-based telepharmacy service models are shown in Figure 4.

#### Service delivery models in community pharmacies

In community pharmacy settings, telepharmacy services regulated by the Pharmacy Council of Thailand allow medications to be delivered directly to patients’ homes, provided that professional practice standards and certified platform requirements are met.

The typical service process begins when patients initiate a remote consultation with a pharmacist through approved applications and complete identity verification. During the consultation, pharmacists provide medication counselling and dispense medicines in accordance with professional standards. Following verification and payment, medications are delivered to the patient's residence via registered logistics providers. Pharmacists subsequently conduct follow-up procedures to monitor symptoms, medication adherence, and potential adverse drug reactions, thereby ensuring continuity of care and therapeutic effectiveness.

### 3.3 Evidence on telepharmacy service effectiveness as policy-informing data

In addition to regulatory reforms and service delivery models, evidence on service effectiveness provides important policy-informing insights into how telepharmacy functioned in routine practice during the COVID-19 pandemic. As telepharmacy was introduced in response to the COVID-19 pandemic in Thailand, several studies compared outcomes among patients with chronic diseases receiving conventional care with those following the implementation of telepharmacy or medication delivery services to assess whether continuity of care could be maintained under these new models of care. The included studies evaluated patients with asthma, epilepsy, HIV infection, hypertension, chronic kidney disease, and, most commonly, diabetes mellitus. Based on the PRISMA-guided study identification process described in the Methods section, this section synthesises the available evidence to illustrate the contribution of telepharmacy to health system resilience.

For studies examining telepharmacy or medication delivery services among patients with chronic diseases in Thailand between 2020 and 2025, a total of 870 records were identified. After removal of 17 duplicate records, 853 records underwent initial title-level screening for broad relevance, during which 761 records clearly unrelated to telepharmacy or medication delivery services were excluded. The remaining 92 records were screened by title and abstract against the eligibility criteria, of which 58 were excluded. Thirty-four full-text articles were assessed for eligibility, and three were excluded because they did not meet the eligibility criteria. In total, 31 studies were included in the review: 14 pharmacist-led telepharmacy studies (Table 2) and 17 studies of telemedicine with medication delivery or medication delivery without pharmacist counselling (Table 3 and Figure 1).

For the purpose of evidence synthesis, the included studies were classified according to the primary model of service delivery and the level of pharmacist involvement. Pharmacist-led telepharmacy referred to services in which pharmacists provided direct teleconsultation, medication review, follow-up, or other pharmaceutical care. Telemedicine with medication delivery referred to services in which physicians and/or other healthcare professionals (e.g. nurses or Village Health Volunteers [VHVs]) provided remote consultation or follow-up before medication delivery, with limited or no direct pharmacist involvement. Medication delivery without pharmacist counselling referred to services in which medications were delivered directly to patients without remote pharmaceutical consultation. Pharmacist-led telepharmacy studies are presented in Table 2, whereas telemedicine with medication delivery and medication delivery without pharmacist counselling models are summarised in Table 3.

**Table 2.** Characteristics and policy-relevant outcomes of pharmacist-led telepharmacy interventions for patients with chronic diseases in Thailand (2020–2025).

Author, year	Disease/Population	Study design (*N*)	Care setting	Type of telecommunication	Policy-relevant outcomes	Implementation context/limitations
**Thavornwattanayong & Nuallaong, 2023**	Asthma	Quasi-experimental pre–post study (*N* = 29)	Regional hospital	Web-based video consultation (TeleHealthRegion7); digital educational materials; home medication delivery	Improved asthma control and adherence (both *P* < 0.01); reduced drug-related problems (DRPs) (*P* < 0.01) and emergency visits; cost savings reported	Regional hospital-based telepharmacy using a real-time video conferencing platform to deliver pharmacist-led consultations; access dependent on smartphones and stable internet connectivity; single-site study; small sample size; short follow-up (3 months); non-randomised pre–post design without control or blinding
**Chomborisut, 2025**	Asthma	One-group pre–post study (*N* = 26)	Community hospital	Video consultation and messaging (LINE Official Account application); digital educational materials; home medication delivery	Improved asthma control (*P* < 0.05) and inhaler technique; reduced DRPs, emergency visits and hospitalisations; improved medication adherence and patient knowledge regarding asthma, medications, and self-care; cost savings reported	Telepharmacy-based pharmaceutical care in community hospital, using the Line Official Account application for pharmacist counseling; required patient access to smartphones and internet; single-site study; small sample size; short follow-up (2 months); non-randomised design without control or blinding
**Lertsinudom et al., 2023**	Epilepsy	Prospective descriptive pre–post study (*N* = 80)	Tertiary hospital	Video consultation (web-based platform and LINE application); home medication delivery	Improved symptom control in a subset of patients; DRPs identified and resolved; adherence support; cost savings reported	University hospital-based telepharmacy using a custom web-based platform with embedded video conferencing for synchronous pharmacist consultations; required smartphone/internet access and patient digital literacy; reliance on patient-reported outcomes; single-site study; small sample size; non-randomised design without control or blinding
**Suntharapot & Sribundit, 2022**	HIV	Randomised controlled trial (*N* = 18 intervention; *N* = 18 control)	Community hospital	Text messaging and follow-up (LINE application); digital reminders; home medication delivery	Improved medication adherence (*P* < 0.001) and CD4 count (*P* = 0.024); no significant difference in viral suppression	ART clinic–based pharmacist-led adherence support using weekly two-way LINE messaging for HIV patients receiving antiretroviral therapy; reliance on patient access to mobile technology; single-site study; small sample size; short follow-up (6 months); no blinding
**Leaungsomnapa et al., 2024**	HIV	Quasi-experimental pre–post study (*N* = 20)	Community hospital	Video consultation (LINE application); home medication delivery	No significant change in viral load or medication adherence	Multidisciplinary telehealth ART follow-up via LINE video calls with pharmacist-led ART consultation and adherence assessment at an antiretroviral clinic; required smartphone and internet access; single-site study; small sample size; non-randomised design without control or blinding
**Rujijanakul et al., 2024**	Chronic kidney disease	Action research pre–post study (*N* = 259)	Community hospitals (provincial network)	Video consultation (Tele–Virtual Health Visit program and LINE application)	Reduced fasting blood sugar (*P* < 0.001) and systolic blood pressure (*P* < 0.002); no significant change in estimated glomerular filtration rate (eGFR); resolution of drug-related problems	Multi-site video consultation implementation across community hospitals using the Tele Virtual Health Visit (Tele VHV) system recording software and LINE; limited staff familiarity with Tele VHV system; human resource constraints; short follow-up (3 months); non-randomised design without control
**Leesakulpisut & Tengmeesri, 2024**	Type I diabetes	Quasi-experimental study (*N* = 18 intervention; *N* = 21 control)	General hospital	Video consultation (LINE application)	No significant difference in readmission rates; reduced hospitalisation costs reported	Pharmacist-led LINE-based video consultations for remote insulin injection technique education implemented in a general hospital; some patients/caregivers experienced difficulties with insulin injection technique; single-site study; small sample size; short follow-up (6 months); non-randomised design
**Sarnjanthuk & Anuratpanich, 2022**	Type II diabetes	Quasi-experimental study (*N* = 40 intervention; *N* = 40 control)	Subdistrict health-promoting hospitals (SHPHs)	Text messaging and follow-up (LINE application)	Improved glycaemic control and lipid parameters (HbA1c, TC, LDL) (*P*<0.05); improved diabetes self-care knowledge	Pharmacist-led LINE-based telepharmacy implemented in a community hospital network using text-based follow-up; required smartphone and internet access; limited network size; small sample size; short follow-up (1 month); non-randomised design without blinding
**Sathanmuang, 2023**	Type II diabetes	Action research pre–post study (*N* = 35)	Community hospital	Video consultation (LINE application)	Reduced fasting glucose and HbA1c; improved medication adherence and insulin-related medication knowledge	Pharmacist-led telepharmacy follow-up implemented in a community hospital using LINE-based video consultation; implementation affected by scheduling coordination between patients and pharmacists and intermittent internet connectivity; single-site study; small sample size; short follow-up (4 months); non-randomised design without control or blinding
**Kulthonkitidej, 2024**	Type II diabetes	Quasi-experimental pre–post study (*N* = 45)	General hospital	Video consultation (iTele program and LINE application); home medication delivery	Reduced HbA1c (*P* < 0.001) but target levels were not achieved; improved medication knowledge; no significant change in medication adherence assessed using the 8-item Morisky Medication Adherence Scale (MMAS-8)	General hospital-based telepharmacy using pharmacist-led remote follow-up via iTele and LINE; access limited for patients without smartphones; single-site study; small sample size; short follow-up (3 months); non-randomised design without control or blinding
**Saralamba et al., 2024**	Type II diabetes	Quasi-experimental pre–post study (*N* = 38)	General hospital	Telephone consultation; home medication delivery	Reduced HbA1c and fasting blood glucose (*P *= 0.003 and 0.022, respectively); improved medication adherence assessed using the Medication Adherence Scale in Thais (MAST) (*P* < 0.001)	Hospital-based telepharmacy program providing pharmaceutical care with remote counselling; relied on consistent phone accessibility; single-site study; small sample size; short follow-up (3 months); non-randomised design without control or blinding
**Fakthongyoo, 2024**	Type II diabetes	Quasi-experimental study (*N* = 100 intervention; *N* = 100 control)	SHPHs	Telephone and video consultations	Reduced fasting plasma glucose and HbA1c within the intervention group (*P* < 0.001); fasting plasma glucose was significantly lower between groups (*P* < 0.001), whereas HbA1c did not differ significantly between groups; improved medication knowledge and adherence	Pharmacist-led telepharmacy implemented across a community hospital network using telephone and video consultations; access limited for patients without smartphones; non-randomised design without blinding
**Buajun, 2025**	Type II diabetes	Quasi-experimental pre–post study (*N* = 34)	Community hospital	Telephone and video consultations	Reduced HbA1c and fasting plasma glucose (*P *= 0.022 and <0.001, respectively); improved medication adherence (MMAS-8) (*P* < 0.001) and diabetes-related knowledge and medication-use knowledge (*P* < 0.001)	Pharmaceutical care delivered via telepharmacy in a community hospital using telephone and video consultations; access limited for patients without smartphones; single-site study; small sample size; short follow-up (6 months); non-randomised design without control or blinding
**Phoosridaow & Ruenhunsa, 2025**	Type II diabetes	Quasi-experimental pre–post study (*N* = 38)	SHPHs	Telephone consultation	Reduced HbA1c (*P* < 0.001); improved insulin injection competency and medication adherence (*P* < 0.05); fasting plasma glucose unchanged	Telepharmacy implemented through a community hospital network using telephone consultations; single-site study; small sample size; short follow-up (4 months); non-randomised pre–post design without control or blinding

Overall, pharmacist-led telepharmacy studies (Table 2) generally reported improvements in chronic disease indicators, including significantly improved asthma control (Chomborisut, 2025; Thavornwattanayong & Nuallaong, 2023), improved epilepsy symptoms (Lertsinudom et al., 2023), improved CD4 counts (Suntharapot & Sribundit, 2022), improved fasting plasma glucose (Buajun, 2025; Fakthongyoo, 2024; Rujijanakul et al., 2024; Saralamba et al., 2024; Sathanmuang, 2023), and improved HbA1c levels (Buajun, 2025; Fakthongyoo, 2024; Kulthonkitidej, 2024; Phoosridaow & Ruenhunsa, 2025; Saralamba et al., 2024; Sarnjanthuk & Anuratpanich, 2022; Sathanmuang, 2023). However, improvements were not consistent across all clinical outcomes. One study reported no significant change in estimated glomerular filtration rate (eGFR) (Rujijanakul et al., 2024), while two HIV studies found no significant improvement in viral load or viral suppression (Leaungsomnapa et al., 2024; Suntharapot & Sribundit, 2022). Across studies, the effectiveness of pharmacist-led telepharmacy appeared to vary according to the outcome assessed, service delivery context, and implementation-related limitations.

In addition to disease control, several studies reported improvements in medication adherence (Buajun, 2025; Chomborisut, 2025; Fakthongyoo, 2024; Phoosridaow & Ruenhunsa, 2025; Saralamba et al., 2024; Sathanmuang, 2023; Suntharapot & Sribundit, 2022; Thavornwattanayong & Nuallaong, 2023). Identification and management of drug-related problems was also a commonly reported component of pharmacist-led telepharmacy models (Chomborisut, 2025; Lertsinudom et al., 2023; Rujijanakul et al., 2024; Thavornwattanayong & Nuallaong, 2023). Furthermore, some studies documented reductions in emergency department visits (Chomborisut, 2025; Thavornwattanayong & Nuallaong, 2023) and potential cost savings for patients and the healthcare system (Chomborisut, 2025; Leesakulpisut & Tengmeesri, 2024; Lertsinudom et al., 2023; Thavornwattanayong & Nuallaong, 2023).

Among studies evaluating telemedicine with medication delivery or medication delivery without pharmacist counselling (Table 3), several disease-specific clinical outcomes were maintained, with no statistically significant changes in blood pressure (Pattarathitinan & Sarinnapakorn, 2023; Rattanapan et al., 2020), viral suppression (Hirandit, 2023), or HbA1c (Pattarathitinan & Sarinnapakorn, 2023; Prakongyot et al., 2024; Tankul, 2022). However, worsening glycaemic control, reflected by increased HbA1c levels, was reported in two studies (Tanjitanont, 2023; Vichian et al., 2023), whereas one study reported improved HbA1c (Boonlert, 2022). In telemedicine-based medication delivery models involving structured follow-up by physicians and/or other healthcare professionals, significant improvements were reported in fasting blood glucose (Buddeekhan, 2023; Khonhan, 2023; Poomiwisate, 2023; Udtamapanya, 2022), HbA1c (Nongkhai, 2024), and blood pressure and LDL cholesterol (Junsom et al., 2025), whereas three studies reported no significant changes in most evaluated clinical outcomes (Sirirachta et al., 2024; Songsermpong et al., 2021; Wangjitchien, 2022).

**Table 3.** Characteristics and outcome patterns of telemedicine with medication delivery and medication delivery without pharmacist counseling service models for patients with chronic diseases in Thailand.

Author, year	Disease/Population	Study design (*N*)	Care setting	Service delivery	Outcome patterns	Implementation context/limitations
Rattanapan et al., 2020	Hypertension	Quasi-experimental pre–post study (*N* = 106)	Community hospital	Prescription refill with postal home delivery without teleconsultation	No significant change in blood pressure; Medication knowledge remained high; high patient satisfaction	Mail-order medication delivery service implemented through a community hospital and its affiliated primary healthcare network to reduce overcrowding; single-site study; small sample size; short follow-up (3 months); no control group or blinding
Hirandit, 2023	HIV	Retrospective cohort study (*N* = 440 intervention; *N* = 105 control)	General hospital	Physician- and nurse-managed mail-order antiretroviral therapy delivery	Improved medication adherence and viral suppression, but did not differ significantly compared with the control group	Home delivery of antiretroviral medications implemented within a general hospital HIV service using postal delivery; retrospective design; no blinding
Songsermpong et al., 2021	Hypertension, Type II Diabetes	Descriptive study (*N* = 8896)	Community hospital network and primary care network	Physician- and nurse-led telemedicine refill service; LINE-based teleconsultation, home monitoring, and medication delivery	Blood pressure control maintained; glycaemic control slightly improved; reduced waiting time and OPD visit	District-wide reorganisation of noncommunicable disease (NCD) services integrating decentralised primary care and telemedicine to maintain continuity of care; descriptive design; single-site study network; short follow-up (7 months)
Tankul, 2022	Type II Diabetes	Retrospective cohort study (*N* = 405 intervention; *N* = 405 control)	General hospital	Postal home delivery of medication refills without teleconsultation	No significant difference in HbA1c between groups	District hospital-based home delivery of diabetes medications implemented within universal coverage system; retrospective design; single-site study; short follow-up (3 months)
Boonlert, 2022	Type II Diabetes	Retrospective observational pre–post study (*N* = 45)	Community hospital	Home medication delivery via village health volunteers (VHVs)	Reduced HbA1c (*P *= 0.001)	Home medication delivery via VHVs to reduce waiting time and service congestion; retrospective design; limited digital literacy among elderly patients; delivery delays; single-site study; small sample size; short follow-up (6 months); no control group or blinding
Wangjitchien, 2022	Type II Diabetes	Quasi-experimental study (intervention vs control) (*N* = 40 intervention; *N* = 40 control)	Subdistrict health-promoting hospitals (SHPHs)	Physician-, nurse-, and VHV-led service with glucose monitoring, LINE/telephone follow-up, and medication delivery	Reduced fasting blood glucose in intervention group (*P* < 0.05); no change in comparison group; high patient satisfaction	Home-based diabetes care with VHV home visits, glucose monitoring, medication delivery, and counselling via telephone and LINE; no between-group comparison; limited digital literacy among elderly patients; small sample size; short follow-up (3 months)
Udtamapanya, 2022	Type II Diabetes	Quasi-experimental study (*N* = 951 participants; *N* = 242 comparison)	Community hospital	Home visits and medication delivery by health officers and VHVs, with physician follow-up	Reduced fasting blood glucose and mean arterial pressure among participants compared with nonparticipants (*P* < 0.05); improved diabetes-related knowledge and self-care behaviours	Home-based diabetes care with home visits and medication delivery via VHVs to reduce hospital visits; limited communication technology; single-site study; short follow-up (3–6 months); non-randomised; no blinding
Buddeekhan, 2023	Type II Diabetes	Quasi-experimental pre–post study (*N* = 48)	Community hospital	Blood glucose and health follow-up via LINE and telephone with home medication delivery	Reduced fasting blood glucose (*P* < 0.001); improved health behaviours; high patient satisfaction	Physician-led home-based diabetes care with LINE/telephone follow-up and home medication delivery; limited staff and resources; variable digital literacy; increased workload; single-site study; small sample size; short follow-up (3 months); no control group or blinding
Vichian et al., 2023	Type II Diabetes, Hypertension	Descriptive study (*N* = 239)	SHPHs	Home-based NCD care using the NCD@Home application for VHV-led monitoring and medication delivery	Controlled HbA1c decreased; blood pressure control remained stable; reduced outpatient visits; no inpatient readmissions within 1 year	Reorganised home visits using the Web Health Manager and the NCD@Home application for health data recording and monitoring, with home medication delivery; descriptive design; budget constraints; increased staff workload; limited internet access; no control group
Khonhan, 2023	Type II Diabetes	Quasi-experimental pre–post study (*N* = 983)	Community hospital	FBS/HbA1c follow-up from medical records with home medication delivery	Reduced fasting blood glucose and HbA1c compared with baseline (*P*< 0.001); reduced hospital congestion and patient travel	Home medication delivery program with multidisciplinary involvement in a community hospital; single-site study; no control group or blinding
Pattarathitinan & Sarinnapakorn, 2023	Type II Diabetes	Retrospective cohort study (*N* = 102 intervention; *N* = 202 control)	Tertiary hospital	Home medication delivery without teleconsultation	No significant differences in HbA1c, blood pressure, or lipid profiles between groups	Physician-directed medication refills with home delivery implemented in a tertiary hospital; retrospective design; short follow-up (6 months)
Poomiwisate, 2023	Type II Diabetes	Quasi-experimental pre–post study (*N* = 150)	Tertiary hospital	Physician-, nurse-, and VHV-led postal medication delivery with telephone follow-up/counseling	Reduced fasting blood glucose and HbA1c (both *P* < 0.001); high patient satisfaction	Physician-directed medication refills using postal delivery with telephone-based follow-up implemented in a tertiary hospital; heavy reliance on nursing staff; single-site study; short follow-up (6 months); no control group or blinding
Tanjitanont, 2023	Type II Diabetes	Retrospective cohort study (*N* = 61 intervention; *N* = 73 control)	SHPHs	Medication delivery via Prescription Delivery System (no physician visit)	Increased HbA1c in delivery group (*P *= 0.003); reduced systolic blood pressure in both groups (*P* < 0.05)	Physician-directed medication refills without in-person visits, with home delivery by VHVs in a community hospital; retrospective design; behavioural factors not controlled; single-site study; small sample size; no blinding
Prakongyot et al., 2024	Type II Diabetes	Retrospective cohort study (*N* = 434 intervention; *N* = 434 control)	Tertiary hospital-led network (Health Promotion Centres)	Physician- and nurse-managed postal medication delivery without teleconsultation	No significant differences in fasting blood glucose or HbA1c between groups	Physician-directed medication refills delivered by postal home delivery; retrospective design; single-site study network; non-randomised; no blinding
Nongkhai, 2024	Type II Diabetes	Action research with pre–post (repeated-measures) evaluation (*N* = 286)	Community hospital	Physician-directed medication refills with VHV home delivery	Reduced HbA1c over study period (*P* < 0.001); reduced outpatient visits; no inpatient admissions reported	Physician-directed refills with home delivery by VHVs and follow-up from medical records; Limited communication and coordination among physicians, nurses, VHVs, and patients.; increased workload; single-site study; non-randomised; no control group or blinding
Sirirachta et al., 2024	Hypertension, type II diabetes, dyslipidaemia	Quasi-experimental pre–post study (*N* = 135)	Tertiary hospital	Physician-led video telemedicine with postal medication delivery	Reduced LDL-C (*P *= 0.009); no significant change in other clinical parameters (BMI, BP, FBS, HbA1c, TG, TC, and HDL-C); reduced travel and in-person visits; high patient satisfaction	Telemedicine consultations with postal medication delivery and record-based follow-up; older patients required assistance with telemedicine; single-site study; no control group or blinding
Junsom et al., 2025	Hypertension	Action research pre–post evaluation (*N* = 124)	Community hospital	Video consultation (Google Meet); remote BP monitoring; lifestyle counseling; home medication delivery	Reduced systolic and diastolic blood pressure (*P* < 0.001); improved dietary behaviours; reduced LDL cholesterol (*P* < 0.001); improved dietary behaviours (*P* < 0.001); reduced waiting time; high patient satisfaction	Telemedicine-based care implemented in a community hospital using video consultations delivered by a multidisciplinary team; single-site study; small sample size; short follow-up (7 months); non-randomised pre–post design without control or blinding

Overall, variability in outcomes was observed across hospital settings, service models, and service delivery contexts, as well as according to the intensity of healthcare professional involvement. Common methodological and implementation-related limitations included constraints related to access to digital devices and internet connectivity, increased workload among healthcare personnel, single-site study designs, small sample sizes, short follow-up durations, and limited use of controlled and randomised study designs.

## 4. Discussion

The COVID-19 pandemic accelerated the global adoption of telehealth solutions, including telepharmacy, and reshaped traditional healthcare delivery models. By leveraging digital technologies, these services enhanced access to care during periods of crisis, particularly in rural and underserved areas (Selvaraj, 2024). Consistent with these global trends, Thailand, previously constrained by strict pharmaceutical regulations, implemented telepharmacy through strong professional self-regulation under the Pharmacy Council, supported by national policy alignment. Telepharmacy evolved into a system-level intervention that complemented telemedicine by improving service efficiency, reducing patient travel and waiting times, and alleviating congestion within healthcare facilities (Health Intervention and Technology Assessment Program Foundation, 2024). Key regulatory instruments, including certified digital platforms, mandatory telepharmacy training, and compliance with the Personal Data Protection Act (PDPA), were designed to ensure patient safety and define professional accountability, while enabling diverse service models with varying levels of integration and pharmacist involvement.

The regulatory evolution of telepharmacy in Thailand reflects a transition from emergency flexibility toward institutionalised governance. Unlike Australia, telepharmacy has primarily evolved as a supplementary mechanism to support rural and remote care, with regulatory frameworks emphasising the primacy of in-person care and established pharmacist–patient relationships (Nott et al., 2024). During the COVID-19 pandemic, many pharmacy-relevant measures (e.g. telehealth medication reviews, e-prescribing, and pharmacist therapeutic substitution) were introduced as emergency or temporary responses but largely remained an adjunct to existing services, particularly for vulnerable and socially isolated patients (Parajuli et al., 2021). In the United States, telepharmacy existed before COVID-19 but expanded rapidly during the pandemic to maintain medication access and reduce infection risk amid social-distancing measures, staffing disruptions, and pre-existing access problems in underserved areas. This occurred within a fragmented, state-based regulatory framework, where many restrictions remained despite temporary federal flexibilities (CMS/HHS reimbursement changes and HIPAA enforcement waivers) that briefly broadened pharmacists’ ability to deliver telehealth services and sustain care (Unni et al., 2021). In contrast, Thailand implemented nationally coordinated regulatory reforms that formally enabled pharmacist-led telepharmacy as part of routine pharmaceutical care. In Japan, similar to Thailand, telepharmacy gained rapid policy attention and underwent regulatory deregulation during the COVID-19 pandemic. However, actual utilisation of the COVID-19 special telepharmacy measure remained very limited, accounting for only 0.51% of 68,849 cases during May–June 2020 (Shoji & Onda, 2024). While telepharmacy has the potential to reduce geographic barriers, evidence from both Japan and Thailand suggests that these benefits are conditional and may be constrained by digital literacy gaps among older adults (Matsumoto et al., 2021). These cross-country comparisons suggest that Thailand's experience provides useful policy lessons for other low- and middle-income countries, particularly regarding how professional self-regulation can facilitate the rapid implementation of telepharmacy within a national health system.

In Thailand, telepharmacy services were largely delivered within the Universal Coverage Scheme (UCS), positioning telepharmacy as a publicly financed digital health service. However, the financing structure remains only partially institutionalised. Key cost components, including medication delivery logistics, digital platform development and maintenance, and the additional workforce time required for remote pharmaceutical care, have not been explicitly incorporated into UCS reimbursement mechanisms, with many costs absorbed by providers or patients, particularly medication delivery expenses. These findings suggest that regulatory enablement alone is insufficient and must be aligned with sustainable financing mechanisms to support long-term integration.

Building on these financing considerations, differences in telepharmacy service models may influence their cost-effectiveness and sustainability within the UCS context. Models integrating remote consultation by both physicians and pharmacists (e.g. Model 1) appear well-suited for patients with stable chronic conditions and may offer favourable cost-effectiveness by reducing hospital visits and optimising medication use, although their reach may be constrained among populations with limited digital literacy or internet access. Models involving community pharmacy collaboration (e.g. Model 5) may improve convenience for patients requiring hospital-based physician care; however, their implementation has been limited by coordination challenges, particularly in medication stock management and system integration. Models combining in-person physician care with home medication delivery and pharmacist consultation (e.g. Model 2) may represent a more sustainable approach within existing healthcare structures, balancing accessibility with continuity of care. Nevertheless, these interpretations remain exploratory, and further research incorporating formal cost-effectiveness analyses and implementation studies is needed to validate these model-specific differences within the UCS context.

Despite these model-specific differences, a significant institutionalisation gap persists between telepharmacy service delivery and sustainable public financing within the UCS system. Addressing this gap will be critical to ensuring long-term scalability and equity. Pharmacist-led telepharmacy primarily functions as a complementary mechanism supporting medication optimisation, adherence monitoring, and continuity of care within physician-led treatment. However, implementation constraints, particularly high patient volumes and workforce limitations, have driven the widespread adoption of delivery-focused models that prioritise access over comprehensive pharmaceutical care. Across the included studies, models involving pharmacist interventions more frequently reported favourable outcomes than those focused primarily on medication delivery. However, given the heterogeneity across studies, these differences cannot be attributed solely to pharmacist involvement. However, the available evidence remains limited by small studies with short follow-up periods, many of which were conducted in single-site settings. Although one randomised controlled trial was identified, most studies were observational or quasi-experimental in design. Many outcomes rely on patient-reported measures, introducing potential bias. While findings suggest benefits in access and selected clinical outcomes, the available evidence remains limited and heterogeneous. These findings should therefore be interpreted with caution, and further well-designed, multicentre studies are needed to support evidence-informed policy and sustainable integration of telepharmacy. Beyond these methodological limitations, the implementation of telepharmacy is shaped by a range of structural and operational challenges affecting scalability, equity, and long-term sustainability, as discussed below.

### 4.1 Challenges and barriers

#### 4.1.1 Technological access and digital literacy

A major obstacle is the digital divide, particularly among the elderly and those residing in rural areas. According to the International Telecommunication Union (ITU), less than 15% of Thais aged 75 years or older had internet access in 2022 (The National Thailand, 2024). This demographic frequently includes patients with chronic conditions requiring ongoing medication management, while those in remote areas often face unstable connectivity, limiting access to telepharmacy services. These findings align with studies in Table 2, which reported reduced reach of telepharmacy due to limited access to smartphones and internet connectivity.

#### 4.1.2 Data privacy and platform usability

While the Pharmacy Council of Thailand has established standards for certified telepharmacy platforms, only a limited number of applications currently meet these requirements. These certified platforms often have complex user interfaces due to built-in security and identity verification features. As a result, both patients and providers frequently rely on more familiar – but less secure – platforms such as LINE or Facebook Messenger. This raises significant concerns about patient confidentiality and data security, particularly under the PDPA. Balancing usability and compliance remains a persistent challenge in telepharmacy implementation.

#### 4.1.3 Suitability for complex medical conditions

Telepharmacy is not universally appropriate for all patient populations. For patients with complex or unstable chronic conditions such as dementia or epilepsy, effective care may require direct observation or in-person assessment. The epilepsy study (Lertsinudom et al., 2023) highlighted limitations in evaluating long-term symptom progression, suggesting that telepharmacy may be inadequate for conditions requiring frequent clinical reassessment and direct patient monitoring.

#### 4.1.4 Communication and counselling effectiveness

Miscommunication related to medication use represents a notable risk in telepharmacy services. The effectiveness of communication and counselling remains a key challenge, as interactions conducted via messaging applications or online platforms may be prone to misunderstanding. The virtual format can limit nonverbal communication and interactive education, particularly among individuals with low health literacy, older adults, or those with cognitive limitations. As a result, concerns persist regarding the quality of remote counselling, which may be less comprehensive or less effective than in-person interactions.

#### 4.1.5 Legal and documentation challenges

The transition from in-person to digital care introduces additional complexities in documentation and legal compliance. Pharmacists are required to maintain accurate and comprehensive records of all telepharmacy activities, including patient consent, the provision of medication counselling, ongoing monitoring and follow-up, and documentation of referrals. However, integration with existing electronic medical record (EMR) systems remains limited, and inconsistencies in digital documentation may disrupt continuity of care and affect legal defensibility.

#### 4.1.6 Workflow integration and pharmacist workload

Integrating telepharmacy into existing healthcare systems may increase pharmacists’ operational workload. Managing remote and in-person services concurrently can strain workflows, particularly in under-resourced settings. Without adequate staffing or system redesign, pharmacists may experience cognitive overload, thereby increasing the risk of medication errors. These challenges are especially pronounced in small hospitals with limited numbers of pharmacists or in independent pharmacy settings.

### 4.2 Prospects and recommendations

Although COVID-19 has been reclassified as an endemic disease in Thailand, telepharmacy services continue to be utilised and further developed as part of routine healthcare delivery.

Building on the structural challenges identified above, the following recommendations aim to support the sustainable and equitable integration of telepharmacy into Thailand's healthcare system:

*a. Investment in technological infrastructure and system interoperability*
Addressing structural limitations in digital access requires targeted investment in both infrastructure and platform development. Expanding internet connectivity in rural and underserved areas, together with the development of standardised and user-friendly telepharmacy platforms, is essential to support broader service adoption. In particular, limited digital access among older adults necessitates context-specific strategies. Leveraging Thailand's Village Health Volunteers (VHVs) as ‘digital mediators’ to assist with technology use and facilitate teleconsultations represents a practical approach to improving access and reducing inequities. In parallel, strengthening interoperability between telepharmacy platforms and existing health information systems within hospital settings is essential to ensure continuity of care. Integrating telepharmacy data with electronic medical records and medication management systems would enable seamless information exchange across care settings, reduce fragmentation, and enhance medication safety. Strategic investment in standardised digital infrastructure and data exchange frameworks is therefore required to support coordinated and sustainable telepharmacy services.
*b. Strengthening regulatory and policy alignment*
Thailand's Drug Act B.E. 2510 requires targeted amendments to align with contemporary telepharmacy practice and reduce legal ambiguity. Three priority areas for legal reform are identified. First, Section 12 (licensing of drug sales) should be expanded to explicitly include online and platform-based dispensing, as current provisions do not clearly recognise digital pharmaceutical services. Second, pharmaceutical advertising provisions under Chapter 11 (Section 88) require updating to address digital marketing, as existing controls are primarily designed for conventional media and do not adequately cover online promotion, social media, or platform-based sales. Third, Section 19 should be revised to clarify that home delivery of medications constitutes part of licensed pharmacy services rather than unlawful sale outside permitted premises. The law should also define eligible drug categories for delivery, restrict high-risk substances (e.g. narcotic drugs), and establish standards for secure distribution and traceability.
*c. Financing support and integration within Universal Health Coverage*
The sustainability of telepharmacy in Thailand requires clearly defined reimbursement mechanisms within the Universal Coverage Scheme (UCS), as both providers and patients face financial burdens. From the provider perspective, telepharmacy entails initial and ongoing costs, including digital platform development and maintenance, information technology infrastructure, internet connectivity, and additional pharmacist workload. From the patient perspective, access to telepharmacy may require smartphones, internet or mobile data, and medication delivery fees, which may disproportionately affect rural and low-income populations. Given these additional costs associated with remote service delivery, a differentiated financing approach may therefore be appropriate. In community pharmacy settings, where telepharmacy is delivered as a standalone service, a fee-for-service model could ensure adequate compensation for pharmacist-led activities. In contrast, hospital-based telepharmacy, which is typically integrated within telemedicine, may be better supported through bundled payments that cover physician consultation, pharmacist services, and medication delivery within a single episode of care. Such an approach may provide a context-appropriate strategy to support both sustainability and equity in telepharmacy implementation.
*d. Directions for future research*
For future policy refinement and service optimisation, further research is needed to address evidence gaps identified in this review. Study areas should include longitudinal and comparative studies to determine which telepharmacy models are most effective across both hospitals and community pharmacies, assess which configurations yield the greatest health and economic benefit, and evaluate the scalability of telepharmacy services across healthcare facilities.

## 5. Conclusion

This review highlights how the COVID-19 pandemic accelerated the development of telepharmacy in Thailand through coordinated regulatory reforms and diverse service delivery models. While telepharmacy has been integrated into routine care under the Universal Coverage Scheme, available studies suggest feasibility and, in several settings, broadly comparable outcomes to conventional care. Addressing persistent structural, operational, and financing challenges will be essential to ensure its long-term and equitable integration beyond the pandemic context.
